# Relationship of prenatal methylmercury exposure and language/verbal function: a meta-analysis

**DOI:** 10.1186/s12940-025-01228-w

**Published:** 2025-09-29

**Authors:** Leonid Kopylev, Deborah Segal

**Affiliations:** Center for Public Health and Environmental Assessment, Office of Research and Development, US Environmental Protection Agency, 1200 Pennsylvania Ave, Washington DC, NW 20460 USA

**Keywords:** Systematic review, Biomarkers, Neurodevelopment, Methylmercury, Mercury

## Abstract

**Background:**

Developmental neurotoxicity (DNT) is a well-established hazard of methylmercury (MeHg) exposure. Past research on MeHg has highlighted DNT tests of language/verbal function (in particular the Boston naming test (BNT)) as an important aspect of MeHg toxicity.

**Methods:**

We conducted a meta-analysis based on a recent systematic review of MeHg neurodevelopmental dose-response cohort studies published 1998–2025 that reported similarly normed tests of language/verbal function. Meta-analyses were based on recent studies using maternal blood biomarkers or cord blood biomarkers converted into maternal blood biomarkers.

**Results:**

For the BNT with or without cues, analysis (based on 2 studies (3 populations)) results were adverse, but not statistically significant. For the similarly normed language/verbal tests, decrements were statistically significant [-0.0085 95% (-0.0167; -0.0003) per MeHg µg/L maternal blood (based on eight studies)]. Results of a fill and trim sensitivity analysis were similar in the size of the effect to the original results. The two studies with sex-specific results indicated that boys appeared to be more sensitive to MeHg-related language/verbal function decrements when compared with girls.

**Conclusions:**

Although most of the individual study results of language/verbal function were not statistically significant, the meta-estimate showed a statistically significant decrement in language/verbal function in children due to prenatal MeHg exposure.

**Supplementary Information:**

The online version contains supplementary material available at 10.1186/s12940-025-01228-w.

## Introduction

A long history of international research has demonstrated that exposure to methylmercury (MeHg), an organic form of the heavy metal mercury (Hg), is associated with developmental neurotoxicity (DNT) [1, 2]. MeHg is also the most common form of Hg to which humans are exposed [3]. It has been previously noted that approximately 3% of the U.S. population has MeHg blood concentrations above 5.8 µg/L, the concentration corresponding to the U.S. EPA (Environmental Protection Agency) recommended limit to prevent harmful health effects if exposed for a lifetime [4]. Most human exposure to MeHg occurs via consumption of contaminated fish or seafood [5], but other foods also significantly contribute to human exposure [6].

The NRC [7] developed MeHg exposure limits based on results of the Boston Naming Test (BNT), which assesses language/verbal ability–a component of cognitive function. Cognitive function tests administered to children are frequently used as measures of DNT resulting from prenatal exposure to chemicals and range from domain specific tests, such as the BNT, to umbrella tests that attempt to quantify overall cognitive ability, such as IQ tests. The existing EPA Reference dose (RfD) [1] was derived from results of several DNT tests (in particular the BNT) administered to 7-year-old children from the Main Faroe cohort [8]. Since then, a number of publications have evaluated language/verbal function effects of MeHg exposure in different populations and at different ages. There are existing meta-analyses for MeHg exposure and broader measures of cognition [9–11]. However, to our knowledge, there has been no meta-analysis of MeHg exposure and language/verbal function. To fill this gap, using the results of our own systematic review of peer-reviewed literature, we conducted a meta-analysis of MeHg exposure and language/verbal function based on tests that were similarly normed (e.g., mean 100 and SD 15). In addition, we performed separate meta-analyses of results from the BNT, because the BNT is not normed. The BNT was administered both with or without cues, because when the test-taker can’t provide a correct response without cues, providing cues helps differentiate between degrees and types of language/verbal impairments. The BNT is scored based on the number of correct answers and the number of cues given. A recent publication [12] reported that the modeling of results based on cord or maternal blood biomarkers is more sensitive to the DNT effects of MeHg exposure resulting in larger risk estimates than modeling results based on maternal hair biomarkers. We, therefore, only included studies using maternal or cord blood biomarkers as MeHg exposure measures in our meta-analyses. However, sensitivity analyses were conducted that included hair biomarker studies.

## Methods

### Literature review methods

A comprehensive literature search was designed to identify epidemiology studies investigating the relationship of MeHg exposure and language/verbal function that were not included in the U.S EPA [1] assessment as part of a broader systematic review described elsewhere [13]. The approach is summarized briefly here (see inclusion/exclusion criteria specified for populations, exposures, comparators, and outcomes (PECO) in Supplemental Table 1). The literature search covered the period of January 1, 1998, to January 25, 2025, utilizing PubMed, Web of Science, Toxline, Science Direct, and SCOPUS. After removing duplicate references, literature search results were filtered in SWIFT Review software by applying specific epidemiology and dose-response search strings [14]. The filtered studies were uploaded into DistillerSR software (DistillerSR Inc. Version 2.35 https://www.distillersr.com/) for manual title/abstract and full-text screening by two independent reviewers to identify studies with quantitative dose-response data for DNT effects of MeHg. In cases of a conflict between primary reviewers, a third reviewer was involved in the resolution.

We evaluated the selected studies for risk of bias and sensitivity using the EPA Integrated Risk Information System (IRIS) approach [15], which assesses the following study domains: participant selection, exposure measurement, outcome ascertainment, confounding, analysis, selective reporting, and study sensitivity. Each study domain was rated as: *good*,* adequate*,* deficient*,* or critically deficient*. Because analytical chemistry methods for measurement of MeHg biomarkers require specialized expertise and are subject to more complications than analyses of other metals [16], studies were first evaluated for exposure measurement quality. Studies receiving a *good* or an *adequate* exposure measurement rating were then evaluated for other study domains. In addition, detailed outcome domain guidance was used to evaluate DNT test measurements [17]. All studies used in these meta-analyses only presented results using total Hg (THg). However, THg is a strong predictor of MeHg in blood or hair in exposed populations [18]. An overall study confidence rating (*high*,* medium*,* low*,* or uninformative*) was assigned by considering the ratings for each domain [15]. Each study was evaluated for risk of bias by at least two independent reviewers. Evaluations were documented in the EPA Health Assessment Workspace Collaborative (HAWC) software [19], a web-based content management system used to develop literature inventories. While the full systematic review included all exposure windows for DNT outcomes in humans [13], the verbal/language function meta-analyses only included a subset of these studies specifically focused on prenatal exposure and relevant tests at any age (see refined inclusion/exclusion criteria for these meta-analyses specified in PECO criteria in Supplemental Table 1).

Only studies classified overall as *high* or *medium* confidence were considered for these meta-analyses, as any limitations identified in these studies are considered unlikely to have a significant impact on the study results. These studies underwent full data extraction of results in Distiller SR, and the extraction results were checked for accuracy. Extracted data included study population demographics, exposure measures (including information on biomarker type and data transformations performed), language/verbal function tests, participant ages, and dose-response information (β’s and corresponding confidence intervals (CI)). Fully adjusted model results were considered when authors presented multiple ways to adjust for covariates.

### Exclusions

Two BNT studies [20, 21] that only reported a composite score (i.e., the sum of the with cues score and without cues score) were excluded because we were interested in the specific effects measured by the separate scores. Another BNT study [22] reported scores for 22-year-olds, but the rest of the included BNT studies reported scores for children 7–12 years of age. Because 22-year-olds had much higher mean scores than those aged 7–12 years, the BNT results of the 22-year-olds were excluded from the meta-analysis. In addition, three studies used outcome measures different from other studies: two studies used the percent of test score standard deviation as an outcome measure [23, 24], and one study used the square root of the BNT score as an outcome measure [25], so these dose-response analyses were excluded. The latter study [25], however, provided scores for another language/verbal test, the Clinical Evaluation of Language Fundamentals, fifth edition (CELF-5 [26]), which were included in the meta-analysis of the normed tests (mean 100 and SD 15). The remaining excluded studies are as follows: three [23, 25, 27] that combined results from several language/verbal tests, three [8, 28, 29] that used subtests that are not normed to a mean of 100 and SD of 15, and two [30, 31] that used raw non-BNT test scores only. Three studies [32–34] administering the MSCA Verbal subtest (mean 50 and SD 10) were excluded as well.

### Data transformations

In some cases, the effect estimates extracted from the epidemiology papers were originally modeled by the study authors after log-transformation of exposure measures using different bases (2, *e*, 10). Using methodology [35], these modeling results (i.e., betas and confidence intervals (CIs)) were re-expressed to approximate the results that would have been obtained without log-transformation in order to facilitate comparisons across studies. Quantitative results (betas and standard errors) were then converted to the common maternal blood biomarker equivalents by using standard conversion factors (1.7 for the ratio of µg/L MeHg in cord blood to µg/L MeHg in maternal blood and 250 for the ratio of ng/g MeHg in maternal hair to µg/L MeHg in maternal blood) [36, 37].

### Statistical methods

All analyses were performed using the R statistical software package *Metafor* version 4.8-0 [38]. A random effects model was used for the meta-analyses, as it is reasonable to assume that true study-specific estimates vary across studies (or subpopulations within the studies). Summary estimates and the 95% CIs are reported for each analysis. The Cochran’s Q-test and I^2^ (ratio of total heterogeneity to total variability) was used to evaluate heterogeneity in the data. To assess possible publication bias, funnel plots were evaluated visually and with Egger’s test. For the sensitivity analysis of publication biases, the trim and fill procedure was used [39]. Five additional sensitivity analyses were conducted: one included hair biomarker studies, a second excluded studies with Bayley Scales of Infant Development (BSID) measures as those are administered to very young children, a third considered different studies of the same (sub)populations (PHIME and Project VIVA cohorts), a fourth considered a leave one out analysis, and a fifth considered only studies using cord blood biomarkers.

## Results

One cohort study [40] included in these meta-analyses was rated as *high* confidence based on the study evaluation. The rest (*N* = 12) were rated as *medium* confidence.

**Fig. 1:**
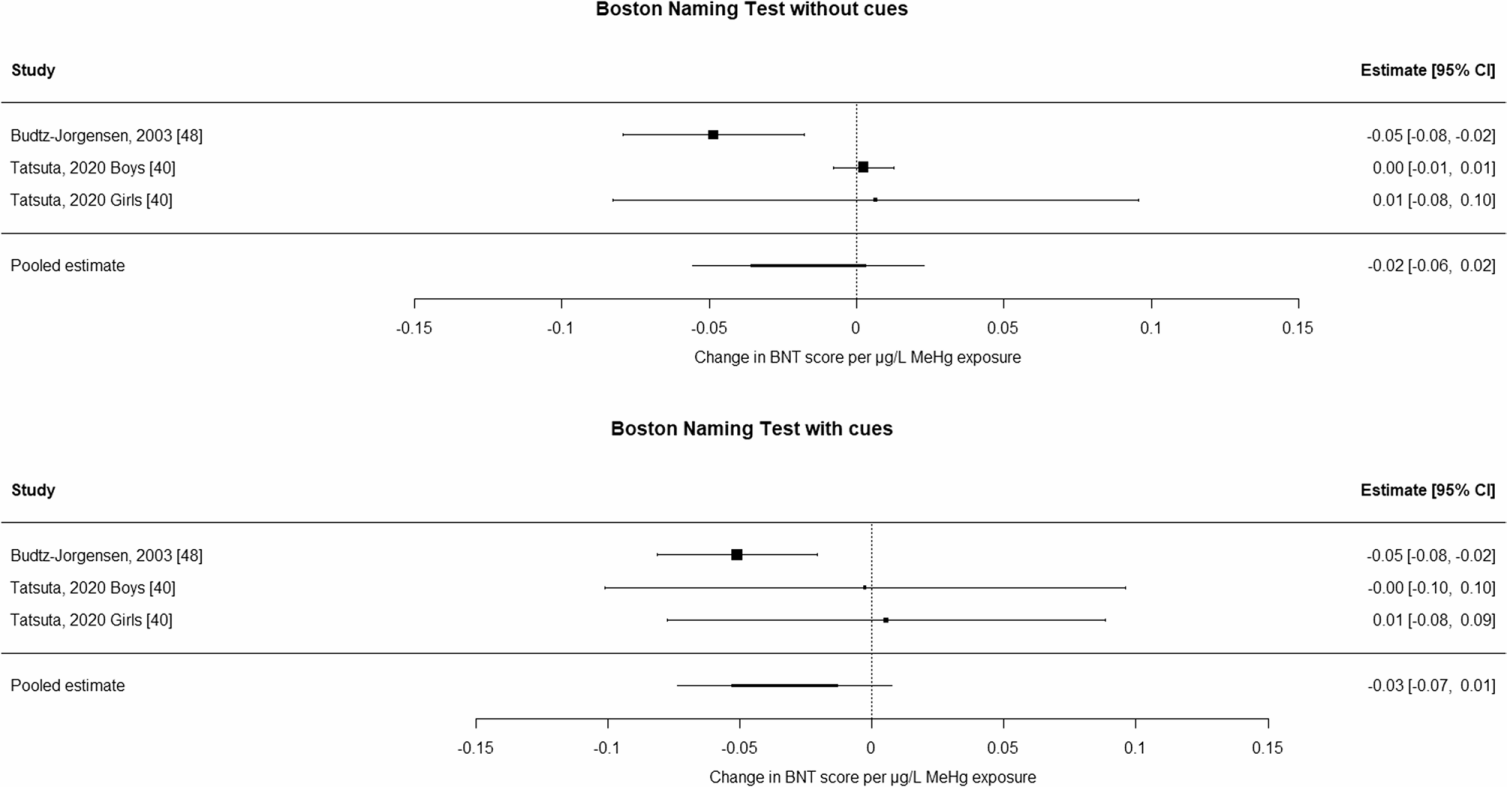
Meta-analysis ofBoston Naming Test without (top panel) and with (bottom panel) cues

Summaries of the data extraction for each study are provided in Table 1 and Supplemental Table 2. For the BNT without cues (Fig. 1 top), the meta-estimate was not statistically significant [−0.016 (−0.056; 0.023) per µg/L of maternal blood MeHg]. The Cochran’s Q test for heterogeneity was significant (*p* = 0.008) and I^2^ was 77%. Egger’s test for publication bias was not significant (*p* = 0.92). Including one study [42] that used hair biomarkers in the sensitivity analysis resulted in a slightly less adverse beta [−0.011 (−0.036, 0.014) per µg/L of maternal blood MeHg]. Results for the BNT with cues (Fig. 1 bottom) were more adverse, but also not statistically significant [−0.033 (−0.074, 0.007) per µg/L of maternal blood MeHg]. Neither Cochran’s Q test for heterogeneity or Egger’s test for publication bias were significant (*p* = 0.33 and *p* = 0.15), and I^2^ was 25%.

**Table 1:** Demographic, biomarker, and modeling results (re-expressed and converted to maternal blood) for BNT and language/verbal function tests

Cohort Location Stratification	Author Year	*N*	Mean age at testing	Biomarker Transformation of exposure	Average Exposure (µg/L maternal blood)	Test	language/verbal function Beta (SE) per µg/L maternal blood
Boston Naming Test							
Faroe one*	Budtz-Jorgensen et al. 2003 [41]	913	7y	Cord Blood log_10_	17.8	No cues Cues	−0.0485(0.0156) −0.0512(0.0155)
Seychelles Main	LaLonde et al. 2020 [42]	533	9y	Maternal hair None	27.2	No cues	−0.0015 (0.007)
Tohoku Japan boys	Tatsuta et al. 2020 [40]	148	12y	Cord Blood log_10_	9.2	No cues Cues	0.0024 (0.0517) −0.0026 (0.0504)
Tohoku Japan girls	Tatsuta et al. 2020 [40]	141	12y	Cord Blood log_10_	9.1	No cues Cues	0.0065 (0.0454) 0.0053 (0.0424)
Language/verbal function tests							
Canada Nunavik	Jacobson et al. 2015 [43]	253	11y	Cord blood ln	12.8	WISC-IV Verbal	−0.0084 (0.0042)
MIREC Canada boys	Packull-McCormick et al. 2023 [44]	255	40 m	Maternal blood log_2_	0.68	WPPSI-III Verbal	−0.97 (2.07)
MIREC Canada girls	Packull-McCormick et al. 2023 [44]	269	39 m	Maternal blood log_2_	0.60	WPPSI-III Verbal	−0.19 (1.26)
MOCEH South Korea	Jeong et al. 2017 [45]	445	5y	Maternal Blood log_2_	3.14	WPPSI-R Verbal	−1.10 (0.396)
PHIME Slovenia Croatia e4**	Snoj Tratnik et al. 2017 [46]	51	18 m	Cord Blood ln	4.0	BSID-III Language	−1.71 (1.62)
PHIME Slovenia Croatia e2e3**	Snoj Tratnik et al. 2017 [46]	232	18 m	Cord Blood ln	3.4	BSID-III Language	−0.476 (1.19)
PHIME Slovenia Croatia	Trdin et al. 2019 [58]	241	18 m	Cord Blood ln	3.1	BSID III Language	0.300 (0.558)
PHIME Italy	Valent et al. 2013 [47]	378	18 m	Cord Blood ln	3.1	BSID-III Language	0.16 (0.213)
Project Viva USA	Oken et al. 2008 [48]	341	3y	Maternal Blood None	3.8	PPVT	−0.4 (0.179)
Project Viva*** USA	Oken et al. 2016 [49]	872	7.9y	Maternal Blood None	4.0	K-BIT-2 Verbal	0.07 (0.145)
Seychelles Main	LaLonde et al. 2020 [42]	533	9y	Maternal Hair None	27.2	WISC III Verbal	−0.0015 (0.007)
Seychelles Nutrition 2	Strain et al. 2021 [25]	1200	7y	Maternal Hair None	15.6	CELF-5	−0.063 (0.12)
Tohoku Japan boys	Tatsuta et al. 2020 [40]	148	12y	Cord Blood log_10_	9.2	WISC-IV Verbal	−0.052 (0.130)
Tohoku Japan girls	Tatsuta et al. 2020 [40]	141	12y	Cord Blood log_10_	9.1	WISC-IV Verbal	0.059 (0.097)
USA World Trade Center	Lederman et al. 2008 [50]	107	4y	Cord blood ln	2.32	WPPSI-R Verbal	−0.532 (0.245)

*BSID-III* Bayley Scales of Infant and Toddler Development [51], *CELF-5 *Clinical Evaluation of Language Fundamentals fifth edition [26], *K-BIT-2 *Kaufman Brief Intelligence Test second edition [52], *PPVT *Peabody Picture Vocabulary Test [53], *WISC-III *Wechsler Intelligence Scale for Children, third revision [54], *WISC-IV *Wechsler Intelligence Scale for Children, fourth revision [55], *WPPSI-R *Wechsler Preschool and Primary Scale of Intelligence, revised edition [56], *WPPSI-III *Wechsler Preschool and Primary Scale of Intelligence–3rd Edition [57], *ln *Natural logarithm, *Y *Years, *M *Months

* Article authors recommended using this analysis, because this analysis included adjustment for an additional covariate compared to the original analysis [8]

**e2e3; *e4* are *Apoe* genotype alleles

*** Data communicated by the author

**Fig. 2:**
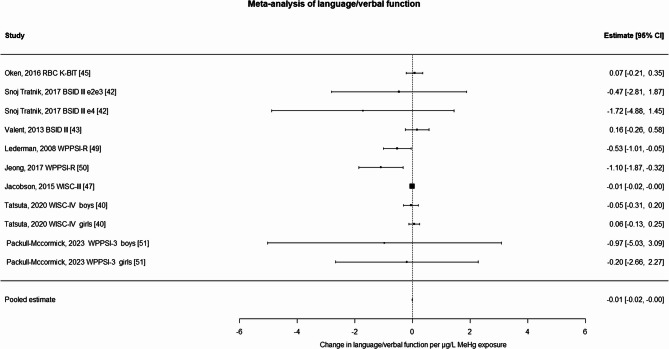
Meta-analysis of similarly normed language/verbal function tests

For language/verbal function tests (Fig. 2), the meta-estimate was statistically significant [−0.0085 (−0.0167; −0.0003) per µg/L of maternal blood MeHg] with non-significant results for the Cochran’s Q test for heterogeneity (*p* = 0.13), I^2^ = 0, and Egger’s test for publication bias (*p* = 0.059). After using the trim and fill procedure [39], the Egger’s test p-value was *p* = 0.77 and the procedure resulted in essentially the same estimate [−0.0082 (−0.16,0) per MeHg µg/L maternal blood], but with statistically significant heterogenicity (*p* = 0.012). Adding two studies from the Seychelles cohorts [25, 42] that used hair biomarkers resulted in a less adverse meta-estimate [–0.0067 (−0.014, 0.003) per MeHg µg/L maternal blood]. Investigating sensitivity to results of testing of younger kids by removing two BSID estimates from the PHIME cohort [46, 47] or replacing one study from the PHIME cohort [46] with a different analysis from the same population [58] did not noticeably change the results. However, replacement of one study from the Project Viva cohort population [49] with a smaller subset of the population [48] amplified the decrement [−0.165 (−0.377, 0.046) per MeHg µg/L maternal blood]. The latter smaller study is in younger children, so results of the former larger study are included in the main analysis. Leave one out analysis showed that for all studies except [43] the results were essentially unchanged, but the removing of study [43] amplified the decrement − 0.13 (−0.38, 0.12) per MeHg µg/L maternal blood. Results of the analysis of studies using cord blood biomarkers were very similar to the overall analysis: − 0.0086 (−0.0166; −0.0002) per MeHg µg/L maternal blood.

## Discussion

We evaluated the impact of prenatal MeHg exposure on language/verbal function by conducting three meta-analyses: the first two considered two studies (3 populations) reporting scores on the BNT without and with cues, and the third considered the results of eight studies using language/verbal tests that were normed to a mean of 100 and SD of 15. The BNT meta-analyses are based on a very small set of studies and resulted in adverse, but not statistically significant, estimates. However, results of the meta-analysis of the normed language/verbal tests showed statistically significant adverse results.

The major strength of these analyses of language/verbal function is that they include the results of a large and variably exposed population, with some subpopulations having lower exposures compared to the general population. The meta-analysis of non-BNT language/verbal function tests derives estimates from eight cohorts of children ranging in age from three to twelve years in Asia, Africa and the North America. 

Given the results observed on decreased sensitivity of hair biomarkers compared to blood biomarkers [12], we considered only cord and maternal blood biomarker studies for the main analysis of language/verbal function. As expected, sensitivity analyses that included maternal hair biomarker studies resulted in attenuated adverse associations between MeHg and language/verbal function.

We used standard conversion factors to convert between cord blood and maternal blood, but these conversion factors can vary and may not be the same for the populations with different dietary patterns. Unlike the rest of the populations in this analysis which are exposed to methylmercury via fish consumption, the population from Arctic Quebec [43] is exposed mostly from consuming beluga whale meat [59]. The ratio between maternal and cord exposure is increased with an increase in the exposure via sea mammal consumption [60]. This may partly explain differing adversity results when study [43] was excluded in a sensitivity analysis. On the other hand, sensitivity analyses of cord blood biomarker studies had similar results to the overall analysis.

Currently, there is not enough data to conduct sex-specific quantitative analyses (only two studies present sex-specific data and, in the rest of the studies, the proportions of each sex were close to 50%). Nevertheless, the sex-specific results found in our systematic review indicated that boys might be more sensitive to the effects of prenatal exposure to MeHg on language/verbal function compared with girls [40, 44]. More sex-specific data is needed to explore this potential sensitivity.

All of the studies in these meta-analyses presented results for total mercury (THg) as a surrogate of MeHg exposure in reported language/verbal function modeling results (a study [58] used in the sensitivity analyses was the only one presenting results for both THg and MeHg biomarkers). For exposed populations, THg has been generally shown to be a reliable predictor of MeHg in both maternal hair and maternal/cord blood [18, 47, 61]. There are also no established methodologies to convert measurements of postnatal biomarkers into maternal or cord biomarkers or to ingested doses. Thus, these analyses were limited to studies that used prenatal exposure measures for modeling MeHg-DNT effects.

These analyses used a methodology [35] for re-expression that provides an estimate of the results of a model using untransformed exposure measurements based on the results of a model with log-transformed exposure measurements. A publication [62] compared the method used here with two other re-expression methodologies and concluded that all three options usually resulted in biased results. Despite this potential bias, the original and re-expressed results were statistically consistent in their analyses for typical study sizes (< 5,000), which includes all those used in these analyses. Still, this presents a limitation for these analyses that could be addressed if study authors routinely provided results using untransformed exposure, as log-transformation is often not needed [63].

Overall, our results suggest that prenatal exposure to MeHg leads to deficits in language/verbal function. However, the meta-analyses included a small number of studies. Therefore, additional studies on MeHg exposure and language/verbal function are warranted.

These meta-analyses provide an updated assessment of the association between MeHg exposure and language/verbal function, which may help to inform future risk assessment and risk management decision-making. Although most individual studies of language/verbal function are not statistically significant, results of these meta-analyses indicate that language/verbal function is decreased as the result of prenatal exposure to MeHg.

## Supplementary Information

Supplementary Material 1.

## Data Availability

All data generated or analyzed during this study are included in this published article and its supplementary information files.
