# Supplementary material for: Relationship of prenatal methylmercury exposure and language/verbal function: a meta-analysis
Source: Environ Health. 2025 Sep 29;24:68. doi: 10.1186/s12940-025-01228-w (PMC12481983; doi:10.1186/s12940-025-01228-w)
Supplement: Supplementary file 1 — Supplementary Material 1. [file 12940_2025_1228_MOESM1_ESM.pdf]

SUPPLEMENTAL MATERIALS

PECO element	Evidence
Populations	Human populations exposed during life stages ranging from gestation through adolescence.
Exposures	Any quantitative exposure to MeHg based on biomonitoring data in hair and blood: either direct MeHg measurements or measurements of total mercury (not other forms of mercury, e.g., mercury salts). **Only prenatal exposure is considered.**
Comparators	Referent within-study populations exposed to lower levels of MeHg or total mercury. Quantitative results of the comparisons must include sufficient detail of estimates and variability (e.g., regression coefficients presented with a statistical measure of variation).
Outcomes	DNT outcomes measured at any age including, but not limited to, tests or measures of cognition, motor function, behavior, vision, and hearing. **Only language/verbal function outcomes are considered**

Supplemental Table 1. Overall systematic review populations, exposures, comparators, outcomes (PECO) criteria for epidemiology dose-response data used in the ongoing broader systematic review [13]. Restricted criteria used only in these meta-analyses are bolded.

Cohort<br>Location<br>Stratification	Author<br>Year	N	Published data			Test	Maternal blood data
			Beta<br>(SE or 95% CI or p)	Units; biomarker	Source of statistical estimates		Beta (SE) of change per µg/L maternal blood
				BNT			
Faroe one*	Budtz Jorgensen et al. 2003 [41]	913	-1.61 (p=0.002)<br>-1.70 (p=0.001)	$\log_{10}$ (µg/L)<br>cord blood	Table 1	No cues<br>Cues	-0.0485 (0.0156)<br>-0.0512 (0.0155)
Seychelles<br>Main	LaLonde et al 2020 [42]	533	-0.006<br>(SE=0.028)	ppm maternal hair at birth	Table 1	No cues	-0.015 (0.007)
Tohoku<br>Japan boys	Tatsuta et al. 2020 [40]	148	0.478 (p=0.790)<br>0.112 (p=0.949)	$\log_{10}$ (ng/g)<br>cord blood	Table 4<br>Model 2	No cues<br>Cues	0.0051 (0.0034)<br>0.0221 (0.0816)
Tohoku<br>Japan girls	Tatsuta et al. 2020 [40]	141	0.149 (p=0.925)<br>0.335 (p=0.821)	$\log_{10}$ (ng/g)<br>cord blood	Table 4<br>Model 2	No cues<br>Cues	0.0153 (0.0646)<br>0.0068 (0.0714)
				Language/verbal function			
Nunavik<br>Canada	Jacobson et al. 2015 [43]	251	-0.15<br>(-0.30, -0.004)	ln (µg/L)<br>cord blood	Table S2<br>Model 3	WISC-IV<br>Verbal	-0.016<br>(0.007)
MIREC<br>Canada<br>boys**	Packull-Mccormick et al. 2023 [44]	214	-0.50<br>(-2.59, 1.59)	$\log_2$ (µg/L)<br>maternal blood	Table S9A, adj. for PCB	WPPSI-III<br>Verbal	-0.45<br>(1.92)
MIREC<br>Canada girls**	Packull-Mccormick et al. 2023 [44]	216	-0.09<br>(-1.23, 1.05)	$\log_2$ (µg/L)<br>maternal blood	Table S9B, adj. for PCB	WPPSI-III<br>Verbal	0.87<br>(1.36)
MOCEH<br>South Korea	Jeong et al. 2017 [45]	445	−2.449 (−4.183, −0.716)	$\log_2$ (µg/L)<br>maternal blood	Table 2<br>Model 2A	WPPSI-R<br>Verbal	-0.76<br>(0.463)
PHIME<br>Slovenia<br>Croatia e4^	Snoj Tratnik et al. 2017 [46]	51	-2.61 (-7.41, 2.20)	ln(ng/g) cord blood	Table 4<br>Model 3	BSID III<br>Language	-1.71 (1.62)
PHIME<br>Slovenia<br>Croatia e2e3^	Snoj Tratnik et al. 2017 [46]	232	-0.59 (-3.42, 2.45)	ln(ng/g) cord blood	Table 4<br>Model 3	BSID III<br>Language	-0.476 (1.19)
PHIME<br>Slovenia<br>Croatia	Trdin et al. 2019 [58]	241	0.71 (−1.88, 3.30)	ln(ng/g) cord blood	Table 6<br>Combined	BSID III<br>Language	0.300 (0.558)

Cohort Location Stratification	Author Year	N	Published data			Test	Maternal blood data
			Beta (SE or 95% CI or p)	Units; biomarker	Source of statistical estimates		Beta (SE) of change per µg/L maternal blood
PHIME Italy	Valent et al. 2013 [47]	378	0.4142 (p=0.4556)	ln(ng/g) cord blood	Table 3 Model 4	BSID III Language	0.160 (0.213)
Project VIVA	Oken et al. 2008 [48]	341	-0.4 (-0.8, -0.1)	ng/g maternal blood	Table 4 adj. additionally	PPVT	-0.4 (0.179)
Project VIVA~	Oken et al. 2016 [49]	872	0.07 (-0.21, 0.36)	ng/g maternal blood	Fig 1 A adj. for fish	K-BIT-2 Verbal	0.07 (0.145)
Seychelles Main	LaLonde et al. 2020 [42]	533	−0.006 (SE=0.028)	ppm maternal hair at birth	Table 1	WISC III Verbal	-0.0015 (0.007)
Seychelles Nutrition two	Strain et al. 2021 [25]	1200	-0.25 (SE=0.48)	ppm maternal hair at birth	Table 2 Model 2	CELF-5	-0.063 (0.12)
Tohoku Japan boys	Tatsuta et al. 2020 [40]	148	-2.585 (p=0.550)	$\log_{10}$ (ng/g) cord blood	Table S3 Model 2	WISC-IV Verbal	-0.612 (0.663)
Tohoku Japan girls	Tatsuta et al. 2020 [40]	141	1.514 (p=0.647)	$\log_{10}$ (ng/g) cord blood	Table S3 Model 2	WISC-IV Verbal	0.343 (0.507)
USA World Trade Center	Lederman et al. 2008 [50]	107	-2.87 (p=0.030)	ln (µg/L) cord blood	Table 5 Full model	WPPSI-R Verbal	-1.141 (0.397)

* Article authors recommended using this analysis, because this analysis included adjustment for an additional covariate compared to the original analysis [8].

**In the MIREC study [44], the number of cord blood measurements below level of detection (LOD) were 25% for males and 34% for females, so only maternal blood results were used because of much lower number of measurements below LOD (8% and 12% correspondingly).

^*e*2; *e*3*e*4 are *Apoe* genotype alleles

~ data communicated by the author

BSID-III--Bayley Scales of Infant and Toddler Development [51]; CELF-5--Clinical Evaluation of Language Fundamentals fifth edition [26]; K-BIT-2--Kaufman Brief Intelligence Test second edition [52]; PPVT--Peabody Picture Vocabulary Test [53]; WISC-III--Wechsler Intelligence Scale for Children, third revision [54]; WISC-IV--Wechsler Intelligence Scale for Children, fourth revision [55]; WPPSI-R--Wechsler Preschool and Primary Scale of Intelligence, revised edition [56]; WPPSI-III--Wechsler Preschool and Primary Scale of Intelligence–3rd Edition [57]. Adj.—adjusted; ln-–natural logarithm.

Supplemental Table 2. Published results for studies modeling the relationship between prenatal exposure to MeHg and language/verbal function, including the source of statistical estimates as well as re-expressed and modeling results converted to maternal blood (µg/L).
